# Polygenic analysis of inflammatory disease variants and effects on microglia in the aging brain

**DOI:** 10.1186/s13024-018-0272-6

**Published:** 2018-07-24

**Authors:** Daniel Felsky, Ellis Patrick, Julie A. Schneider, Sara Mostafavi, Chris Gaiteri, Nikolaos Patsopoulos, David A. Bennett, Philip L. De Jager

**Affiliations:** 1 Center for Translational and Computational Neuroimmunology, Department of Neurology, Columbia University Medical Center, 630 West 168th Street, PH 19 – 302, New York, NY 10032 USA; 2 Department of Neurology, Brigham and Woman’s Hospital, 75 Francis Street, Boston, MA 02115 USA; 3 Department of Neurology, Harvard Medical School, 25 Shattuck Street, Boston, MA 02115 USA; 4 Program in Population and Medical Genetics, Broad Institute of MIT and Harvard, 415 Main St, Cambridge, MA 02142 USA; 5 Department of Statistics, University of Sydney, Camperdown, NSW 2006 Australia; 6 Department of Neurology, Rush University Medical Center, 1653 West Congress Parkway, Chicago, IL 60612 USA; 7 Rush Alzheimer’s Disease Center, Rush University Medical Center, 600 South Paulina Street, Chicago, IL 60612 USA; 8 Department of Statistics, University of British Columbia, 2329 West Mall, Vancouver, BC V6T 1Z4 Canada

**Keywords:** Genomics, Alzheimer’s disease, Microglia, Inflammation, Polygenic score, Innate immunity, Postmortem, Neuropathology, RNA sequencing

## Abstract

**Background:**

The role of the innate immune system in Alzheimer’s disease (AD) and neurodegenerative disease susceptibility has recently been highlighted in genetic studies. However, we do not know whether risk for inflammatory disease predisposes unaffected individuals to late-life cognitive deficits or AD-related neuropathology. We investigated whether genetic risk scores for seven immune diseases and central nervous system traits were related to cognitive decline (n_max_ = 1601), classical AD neuropathology (n_max_ = 985), or microglial density (n_max_ = 184).

**Methods:**

Longitudinal cognitive decline, postmortem amyloid and tau neuropathology, microglial density, and gene module expression from bulk brain tissue were all measured in participants from two large cohorts (the Rush Religious Orders Study and Memory and Aging Project; ROS/MAP) of elderly subjects (mean age at entry 78 +/− 8.7 years). We analyzed data primarily using robust regression methods. Neuropathologists were blind to clinical data.

**Results:**

The AD genetic risk scores, including and excluding *APOE* effects, were strongly associated with cognitive decline in all domains (min *P*_*uncor*_ = 3.2 × 10^− 29^). Multiple sclerosis (MS), Parkinson’s disease, and schizophrenia risk did not influence cognitive decline in older age, but the rheumatoid arthritis (RA) risk score alone was significantly associated with microglial density after correction (t_146_ = − 3.88, *P*_uncor_ = 1.6 × 10^− 4^). Post-hoc tests found significant effects of the RA genetic risk score in multiple regions and stages of microglial activation (min *P*_uncor_ = 1.5 × 10^− 6^). However, these associations were driven by only one or two variants, rather than cumulative polygenicity. Further, individual MS (*P*_one-sided_ < 8.4 × 10^− 4^) and RA (*P*_one-sided_ = 3 × 10^− 4^) variants associated with higher microglial density were also associated with increased expression of brain immune gene modules.

**Conclusions:**

Our results demonstrate that global risk of inflammatory disease does not strongly influence aging-related cognitive decline but that susceptibility variants that influence peripheral immune function also alter microglial density and immune gene expression in the aging brain, opening a new perspective on the control of microglial and immune responses within the central nervous system. Further study on the molecular mechanisms of peripheral immune disease risk influencing glial cell activation will be required to identify key regulators of these pathways.

**Electronic supplementary material:**

The online version of this article (10.1186/s13024-018-0272-6) contains supplementary material, which is available to authorized users.

## Background

Genetic studies have highlighted the shared architecture of susceptibility among inflammatory diseases [1]; however, in unaffected individuals, the significance of this genetic predisposition to immune dysfunction for aging-related processes is unknown. Recent genetic studies of neurodegenerative diseases have also implicated the immune system – particularly the innate component – in susceptibility to aging-related conditions [2], with Alzheimer’s disease (AD) susceptibility being most clearly enriched for variants that influence the expression and splicing of genes expressed in myeloid cells [3, 4]. In the brain, microglia, the resident myeloid cells, have a prominent role, but infiltrating macrophages, B cells, and T cells also contribute to brain pathology in older age. Today, it is not clear whether a predisposition to pathologic inflammatory responses influences the function of organ systems in individuals that are not affected by an autoimmune disease. Here, we evaluate whether a propensity for excessive inflammation that has not manifested itself clinically in one’s life course can influence the likelihood of (1) late-life cognitive deficits, (2) the accumulation of common neuropathology, including amyloid-β (Aβ) and hyperphosphorylated tau, or (3) microglial recruitment and activation.

The genetic architecture of aging-related traits is not yet well understood; although their prevalence in the population and the heterogeneity observed among older individuals suggest that the allelic spectrum influencing susceptibility includes many common variants of modest effect [5–7]. To assess this hypothesis, we used a common strategy in which the effects of validated susceptibility variants are aggregated into a single additive genetic risk score (GRS). While useful as a screening tool, a GRS obscures the intrinsic granularity of the genomic risk landscape; therefore, they need to be complemented with targeted investigations of individual variants, where possible. We evaluated two representative inflammatory diseases for which the genetic architecture of susceptibility has been well described: MS, which targets the central nervous system, and rheumatoid arthritis (RA), for which a recent study of medical claims data from over 8.5 million adults reported an increased relative risk of AD among patients with RA. Further, anti-TNFα therapy for RA may lower this risk [8]. In MS, a neuropathological study reported no significant difference in the presence of AD pathologies compared to controls [9]; however, the prevalence of late-life pathologies and cognitive deficits in genetically-defined populations remains to be characterized.

We also evaluated GRS for four other traits that could influence brain aging and have been characterized genetically: Parkinson’s disease (PD), telomere length, coronary artery disease (CAD), as well as schizophrenia, which may also have an inflammatory component [10]. Specifically, we deployed our GRS in two deeply characterized cohorts of aging individuals, deconstructed polygenic associations to resolve whether an association is driven by selected variants or a broad distribution of variants, and accessed cortical RNA sequence data to further develop our mechanistic understanding of observed genetic effects.

## Methods

### Subjects

Participants in this study were from the Religious Orders Study (ROS) [11] and the Rush Memory and Aging Project (MAP) [12], two cohort studies of elderly populations from the Chicago area conducted by investigators at the Rush Alzheimer’s Disease Center (Rush University Medical Center, Chicago, IL, USA). All subjects were older and recruited free of dementia (mean age at entry 78 +/− 8.7 years), agreed to annual clinical and neurocognitive evaluation, and signed an Anatomical Gift Act allowing for brain autopsy at time of death.

### Genetics and imputation

In total, 1878 subjects were genotyped using the Affymetrix 6.0 Genechip. DNA was extracted from whole blood, lymphocytes, or frozen brain tissue and genotype data underwent standard quality control procedures using PLINK (v1.08), as previously described [13]. Briefly, subjects and variants were filtered based on genotype success rate > 0.95, Hardy-Weinberg Equilibrium *P* > 0.001, and mishap test *P*> 1 × 10^− 9^. After quality control of the initial genotype dataset, 1709 individuals and 750,173 autosomal variants remained. Whole genome imputation was performed using BEAGLE (v3.3.2) [14] and the 1000 Genomes reference panel (phase I haplotypes). To analyze the major histocompatibility complex (MHC) region in detail, a specialized imputation pipeline, SNP2HLA [15], was used. This was necessary given the major contribution of variation in this region to the pathogenesis of immune-related traits analyzed in this study. See Additional file 1: Supplementary Methods for details.

### Postmortem amyloid-β and tau neuropathology

Postmortem neuropathology data were available for up to 985 subjects at time of study. All brains were examined by a board-certified neuropathologist blinded to age and clinical data. Aβ and abnormal tau deposition were measured using immunohistochemistry and automated image processing for total amyloid and paired helical filament tau, and a modified Bielschowsky silver staining technique for neuritic and diffuse plaques, and neurofibrillary tangles, as published previously [16].

### Postmortem microglial count density

A subset of up to 183 brain samples with genomic data were evaluated for the presence of microglia at three stages of activation, based on morphology: stage 1 (thin ramified processes), stage 2 (plump cytoplasm and thicker processes), and stage 3 (appearance of macrophages). For each of four regions (midfrontal cortex, inferior temporal cortex, ventral medial caudate, and posterior putamen), four microglial density scores (total count of microglia/area surveyed) were calculated: stage 1 only, stage 1 + 2 + 3, stage 2 + 3, and stage 3 only [3] (see Additional file 1: Supplementary Methods).

### Cognitive decline

A total of 1601 subjects with genomic data also had longitudinal cognitive performance data available at the time of study. ROS and MAP subjects were both administered 17 cognitive tests annually spanning five cognitive domains: episodic memory, semantic memory, working memory, perceptual speed, and visuospatial ability. Measures of cognitive performance for each domain were calculated by averaging z-scores across tests [11, 12, 17], and rates of cognitive decline were calculated per subject using general linear mixed models of cognitive scores over time, co-varying for age at baseline, years of education, and sex, as described [6].

### Gene expression

#### RNA sequencing and post-processing

RNA was extracted from DLPFC and sequenced on the Illumina HiSeq (50 million paired-end reads of 101 bp each), as described [13]. Expression FPKM values were quantile-normalized, correcting for batch effect with Combat [18]. Paired-end reads were mapped to genes using the Ensemble human genome transcriptomic database (http://www.ensembl.org). Expression QTL (eQTL) analyses were performed in the ROS/MAP sample to ascertain potential mechanisms of pathology-associated gene variants with respect to gene expression [19]. The GTEx portal [20, 21] was used to corroborate eQTL effects.

#### Clustering and module enrichment analyses

Gene modules of co-expressed genes were derived using the SpeakEasy consensus clustering algorithm [22]. In ROS/MAP, SpeakEasy identified 47 mutually exclusive modules with 20–556 gene members (median = 331), several of which have been shown to correlate strongly with pathology, cognition, and cell-type specific markers of gene expression in multiple datasets [23]. Of these modules, five (modules #5, #113, #114, #115, and #116) show substantial enrichment for immune- and microglia-related functions and processes [23]. Immune gene modules were defined based on hypergeometric enrichment for microglia-specific genes (enrichment *p* < 0.0011). These microglia-specific genes were defined based on Olah et al. (2018) [24] as at least four-fold upregulated in human bulk microglia. Module 113 (includes AD genes *CLU*, *SPPL2A*, *SQSTM1*, *MPZL1*, and *ETS1*) has an overlap of 24 microglial genes/313 total module genes (*P* = 0.0024); module 114 (no major AD genes) has an overlap of 24/276 (*P*= 4.5 × 10^− 4^); module 115 (no major AD genes) has an overlap of 33/232 (*P*= 4.1 × 10^− 10^); module 116 (*TREM2*, *INPP5D*) has an overlap of 144/224 (*P*= 5.6 × 10^− 148^); and module 5 (*BIN1*, *PVRL2*) has an overlap of 58/431 (*P* =8.5 × 10^− 16^). See Patrick et al. (2018) for details [25]. As such, expression levels of these five modules were used to benchmark the transcriptional effects of variants in functionally cohesive immune pathways.

### Statistical analysis

#### GRS calculation

GRS were calculated using PLINK (v1.90b) [26] and all other analyses were performed using R (v3.3.3) [27]. We tested eight different GRS in this study: two inflammatory disease scores (MS and RA) and comparator scores that could influence aging-related cognitive decline, including those for AD (including (+) and excluding (−) *APOE*), PD (which frequently includes a dementing illness), CAD (the second most common cause of dementia in older individuals), schizophrenia (previously known as dementia praecox), and telomere length (a marker of biological aging). For each score, lists of genome-wide significant variants were extracted from state-of-the-art genome-wide association studies (listed in Table 1). Individual publications were chosen rather than an aggregate database to limit error due to between-study heterogeneity in outcome definitions, sample characteristics, and statistical methodology. The PLINK --score command was then used to generate average per-allele scores, weighted by each variant’s published effect. Briefly, for each published trait, the number of effect alleles (i.e. those associated with an increase in trait outcome or disease liability) for each genome-wide significant variant was multiplied by its effect size (natural logarithm of the odds ratio or standardized beta coefficient), and these quantities were summed across variants within each subject to generate individual polygenic scores. Default parameters were used for this calculation, and missing genotypes contributed an amount to each score equal to the effect allele frequency in our sample, minimizing potential bias. Nonetheless, imputed genotype quality was high and each GRS calculation was manually inspected to ensure negligible and non-systematic missingness.

**Table 1:** Summary of Studies Used to Derive Polygenic Scores

Trait/Disease	Publication	Total study size (cases/controls)^a^	# of SNPs in score	SNPs with ROS/MAP MAF > 0.1
AD	Lambert et al., 2013 (Nat. Genet.)	25,580 / 48,466	22	18
CAD	Nikpay et al., 2015 (Nat. Genet.)	60,801 / 123,504	63	54
MS	Patsopoulos et al.,2017 (Biorxiv)	47,351 / 68,284	232	196
PD	Nalls et al., 2014 (Nat. Genet.)	13,708 / 95,282	32	27
RA	Okada et al., 2014 (Nature)	29,880 / 73,758	76	67
Schizophrenia	Psychiatric Genomics Consortium, 2014 (Nature)	36,989 / 113,075	106	100
Telomere length	Codd et al., 2013 (Nat. Genet.)	48,423^b^	8	8

^a^Study size represents all subjects analyzed, regardless of study design (i.e. case/control, meta-analysis, and family-based designs) or analysis stage.

^b^The study by Codd et al., 2013 [43] was not a case/control design, as telomere length was evaluated as a continuous outcome. MAF = minor allele frequency. MAP = Rush Memory and Aging Project. ROS = Rush Religious Orders Study

#### Associations of GRS with postmortem pathology and cognitive decline

Associations of GRS with study outcomes were modeled using iterated re-weighted least squares, from the “MASS” R package [27]. The iterated re-weighted least squares regression technique employed here provides effect estimates robust to outliers by assigning weights to each observation and iteratively fitting Huber M-estimators [28]. All models of neuropathology co-varied for age at death, sex, postmortem interval, and the top three EIGENSTRAT principal components [29]. Tests were corrected using Benjamini & Hochberg’s false discovery rate (FDR) procedure [30]. Significant associations (two-sided *P*_FDR_ < 0.05) were analyzed further to determine region and activation stage-specific effects on microglial density. All variants within each significantly associated GRS (with minor allele frequency > 0.1) were then tested individually.

#### Overlap between variants affecting microglial density and gene module expression

To compare effects of risk variants on microglial density vs. effects of the same risk variants on gene module expression, the -log(*P*-value) for each variant’s effect on both phenotypes were multiplied by their allelic directions of effect (+ 1 or − 1) and tested for association using Spearman rank correlations. Thus, whole sets of variants, grouped by the GRS to which they contribute, could be tested for synergistic effects on both microglial density and gene module expression. Correlation coefficients were calculated separately for each GRS and module combination, where positive ρ values indicate a tendency for variants within a given GRS to influence both microglial density and gene module expression in concordant directions. The ranks of these coefficients for the five immune modules were then evaluated for significance by calculating the probability that the lowest of five randomly selected ranks would be equal to the lowest observed rank by chance alone (see Additional file 1: Supplementary Methods).

## Results

### The RA but not the MS GRS is associated with cognitive decline and postmortem neuropathology

Table 2 summarizes the demographic characteristics of the ROS/MAP participants that were included in our analyses. In comparing the GRS to one another, we found a modest correlation between the RA and MS scores (Spearman ρ = − 0.13, *P*_uncor_ = 2.29 × 10^− 8^), as expected given the documented sharing of susceptibility loci between the two diseases (see Additional file 1: Figure S1).

**Table 2:** Sample Sizes and Characteristics for Each Analysis

Phenotype	N	Mean	SD	Min	Max
Neuritic Plaques	985	0.86	0.85	0.00	5.04
Diffuse Plaques		0.73	0.77	0.00	4.61
Neurofibrillary Tangles		0.63	0.76	0.00	6.23
Sex (F/M)		641/344	–	–	–
*APOE* ε4 status (−/+)		723/262	–	–	–
Age at death		89.06	6.39	66.22	108.28
Dx at last visit (CN/MCI/AD/other)		319/237/343/86	–	–	–
PMI		8.57	7.59	0.00	85.08
Total Amyloid	952	4.21	4.20	0.00	19.93
Sex (F/M)		617/335	–	–	–
*APOE* ε4 status (−/+)		697/255	–	–	–
Age at death		88.95	6.38	66.22	108.28
Dx at last visit (CN/MCI/AD/other)		309/230/329/84	–	–	–
PMI		8.52	7.57	0.00	85.08
Total PHF-Tau	946	6.43	7.70	0.00	78.52
Sex (F/M)		615/331	–	–	–
*APOE* ε4 status (−/+)		694/252	–	–	–
Age at death		88.91	6.38	66.22	108.28
Dx at last visit (CN/MCI/AD/other)		312/228/326/80	–	–	–
PMI		8.47	7.56	0.00	85.08
Microglial Density (all regions)	154	191.02	54.88	48.30	348.64
Sex (F/M)		96/58	–	–	–
*APOE* ε4 status (−/+)		117/37	–	–	–
Age at death		89.50	5.15	74.83	101.19
Dx at last visit (CN/MCI/AD/other)		51/41/58/4	–	–	–
PMI		7.36	5.97	2.50	54.50
Cognition	1601	−0.01	0.09	−0.48	0.17
Sex (F/M)		1113/488	–	–	–
*APOE* ε4 status (−/+)	1 594^a^	1186/408	–	–	–
Age at baseline evaluation	1601	86.50	6.81	60.15	108.15
Dx at last visit (CN/MCI/AD/other)		700/357/436/108	–	–	–

Note: All values of N are given for samples that have data for both the specified phenotype and genome-wide genotypes.

^a^*APOE* ε4 status was obtained separately from genome-wide genotypes, so seven samples with cognitive data did not have *APOE* ε4 status data available at time of study, *CN* cognitively normal, *Dx* diagnosis, *F* female, *M* male, *MCI* mild cognitive impairment, *PHF-Tau* paired helical filament tau, *PMI* postmortem interval, *SD* standard deviation

**Fig. 1:**
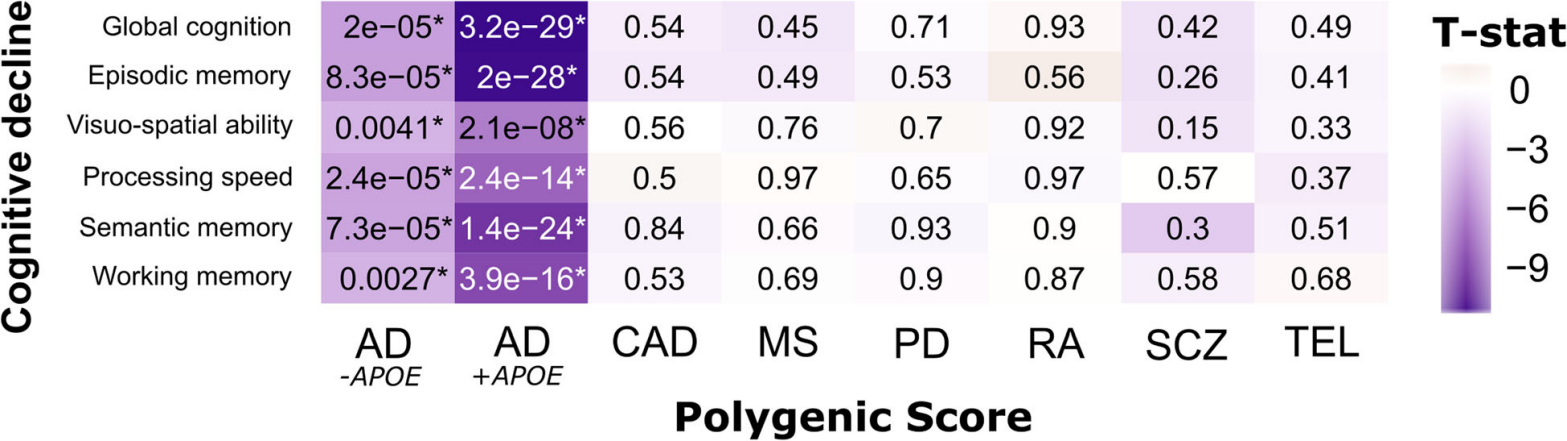
Analysis of GRS vs. cognitive decline slopes (*n* = 1601). Two-sided uncorrected *P*-values derived from robust regression are shown within tiles. Models co-varied for age at initial assessment, sex, years of education, and three EIGENSTRAT principal components. The color scale indicates magnitude and direction of the effect T-statistic. *significant after FDR correction (*P*_FDR_ < 0.05)

**Fig. 2:**
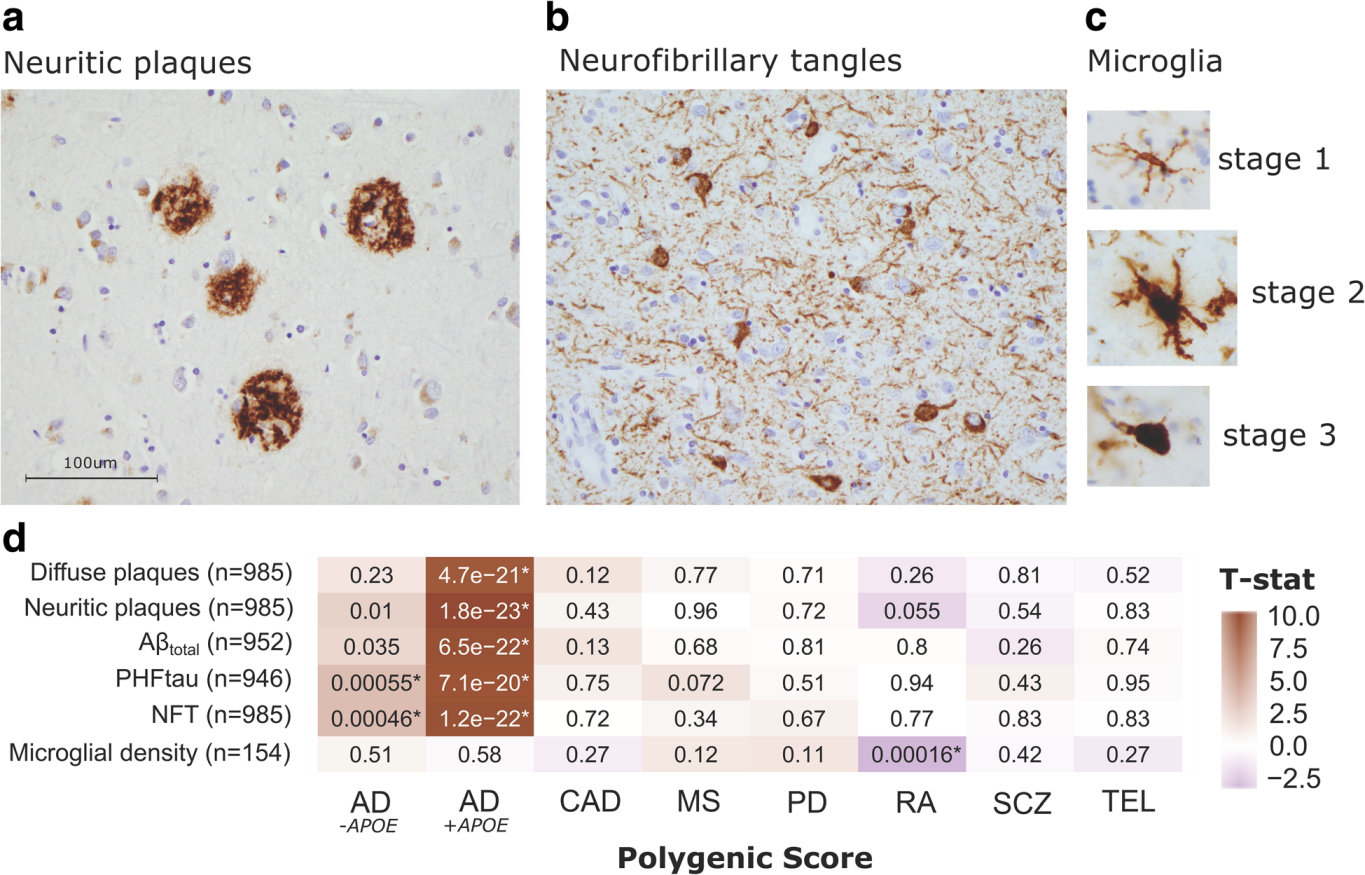
Analysis of GRS vs. aggregate AD-related pathologies and microglial density. Immunohistochemistry images showing (**a**) neuritic amyloid plaques (stained with 4G8), (**b**) neurofibrillary (tau) tangles (stained with AT8), and (**c**) microglia at three stages of activation (stained with CR3–43) in our postmortem tissue samples. (**d**) Two-sided uncorrected *P*-values derived from robust regression are shown within tiles. Models co-varied for age at death, sex, and three EIGENSTRAT principal components. The color scale indicates magnitude and direction of the effect T-statistic. *significant after FDR correction (*P*_FDR_ < 0.05)

**Fig. 3:**
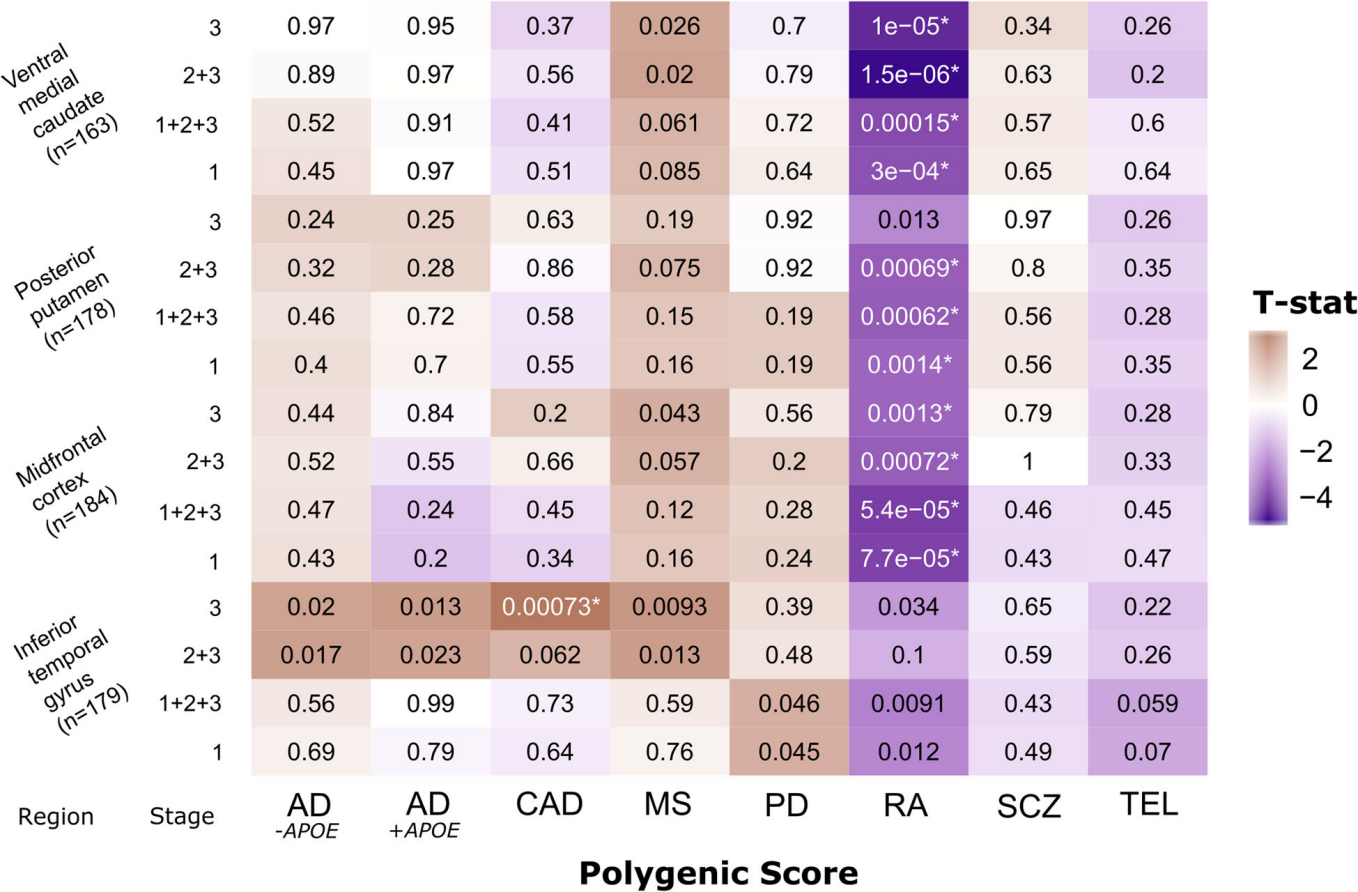
Analysis of GRS vs. microglial densities across four regions for each measured stage of activation. Two-sided uncorrected *P*-values derived from robust regression are shown within tiles. Models co-varied for age at death, sex, and three EIGENSTRAT principal components. The color scale indicates magnitude and direction of the effect T-statistic. *significant after FDR correction (*P*_FDR_ < 0.05)

After FDR correction, both the AD GRS including (AD^+*APOE*^) and excluding (AD^-*APOE*^) the *APOE* ɛ4 risk haplotype were significantly associated with faster decline in all cognitive domains proximal to death (min *P*_*uncor*_ < 1 × 10^− 16^), but no other GRS demonstrated significant associations (Fig. 1). The AD^+*APOE*^ GRS was also strongly associated with both amyloid (4.7 × 10^− 21^ > *P*_uncor_ > 1.8 × 10^− 23^) and tau (7.1 × 10^− 20^ > *P*_uncor_ > 1.2 × 10^− 22^) phenotypes; whereas the AD^-*APOE*^ GRS was only associated with tau measures (5.5 × 10^− 4^ > *P*_uncor_ > 5.5 × 10^− 4^) (Fig. 2). There were no associations of either AD GRS with microglial counts, confirming findings previously reported in these and other cohorts. [5, 31] Across all other scores, only the RA GRS was significantly associated with brain-wide microglial density after correction: an increase in liability for RA was associated with a decrease in microglial density (t_146_ = − 3.88, *P*_uncor_ = 1.6 × 10^− 4^) (Fig. 2). We then accessed our more detailed microglial data and repeated the analyses to test for the effects of each GRS on microglial count density across each brain region and stages of microglial activation. We found that the effect of the RA GRS is widely distributed, being present in multiple brain regions and stages of activation (1.4 × 10^− 3^ > *P*_uncor_ > 1.5 × 10^− 6^). In addition, significant (CAD; t_171_ = 3.44, *P*_uncor_ = 7.3 × 10^− 4^) and suggestive (MS and AD) associations, particularly in relation to the activated stage 3 microglia, were noted in the inferior temporal gyrus (Fig. 3).

### Individual variants drive GRS associations with microglial density

**Fig. 4:**
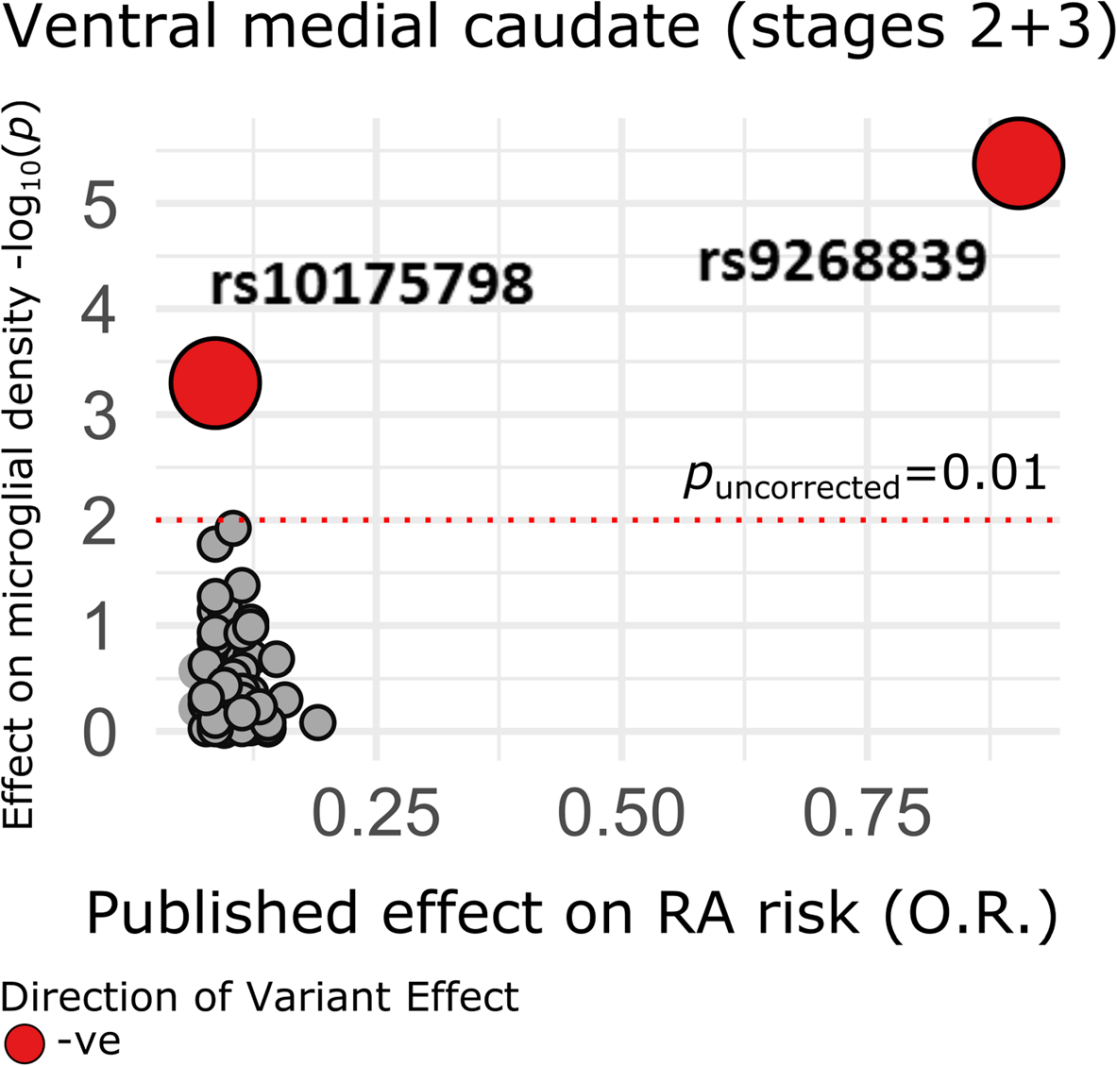
Analysis of individual variants in the RA GRS on microglial density in the ventral medial caudate. Published effect sizes on the x-axis have been transformed using a natural logarithm and oriented in the positive direction to align allelic effects (color denotes direction of effect on microglial density). *P*-values (uncorrected) are two-sided and derived from robust iterated re-weighted least squares regression models, co-varying for age at death, sex, and three EIGENSTRAT principal components

To understand what drives the association of the RA and CAD GRS with microglial phenotypes, we analyzed individual variants contributing to each GRS. For CAD, rs4977574 (chr. 9) alone drove the GRS association, and, for RA, two variants, rs9268839 (chr. 6) and rs10175798 (chr. 2), were responsible (Fig. 4). After removal of these two top RA GRS variants and re-calculation of the RA score, we found no association with total microglial density (*P* = 0.74) nor with any of the region- or stage-specific measures (0.23 > *P* < 0.88). The more strongly associated RA variant, rs9268839, is found in the Human Leukocyte Antigen (HLA) class II region and is also the strongest RA susceptibility variant in Europeans (O.R. = 2.47, C.I._95%_ = [2.39,2.55], *P*_meta_ = 1.5 × 10^− 300^) [32]. Therefore, upweighting of this variant in the calculation of the RA GRS biased its effect on microglial density (Additional file 1: Figure S3). In the CAD GRS, a similar pattern was observed: rs4977574 drives the association and is the strongest hit in the CAD genome-wide association study (O.R. = 1.21, C.I._95%_ = [1.19,1.24], *P*_meta_ = 2.29 × 10^− 98^) [33] (Additional file 1: Figure S4).

In ROS/MAP cortical RNA and GTEx, the top variant influencing microglial density for the RA score (rs9268839, *P*_uncor_ = 2.48 × 10^− 5^) influences HLA class II expression (see Additional file 1: Supplementary Methods). The top CAD SNP, rs4977574, has no cis-eQTL effects in these data but has been reported to influence *VIL2* in blood (*P* = 6.1 × 10^− 6^) [34].

### Variants affecting microglial density also tend to affect expression of immune gene modules

Another approach to exploring the role of GRS in the aging brain involves assessing their effects on the cortical transcriptome of ROS/MAP participants, using the 47 modules of co-expressed genes previously defined in these individuals [23]. Additional file 1: Figure S5A shows the results of the direct association of each gene module’s expression with each GRS. After correction, this analysis yielded no significant results overall; however, 17 correlations exceeded *P*_uncor_ < 0.05. Evaluating gene module expression directly against midfrontal microglia density (stages 1 + 2 + 3), only module #118 had a significant effect after correction (t_94_ = − 3.60, *P*_uncor_ = 5.1 × 10^− 4^), whereby increased expression was associated with a decrease in microglial density (Additional file 1: Figure S5B). To evaluate the intersection of gene variant effects on both microglial density and gene expression, Spearman correlations of variant effects on both outcomes were evaluated within each GRS. Again, no results were significant after correction, largely due to a sparsity of individually significant associations of GRS SNPs with either microglial density or module expression. Nonetheless, our exploratory findings suggest a possible tendency for variants which affect microglial density to also influence immune module expression (See Additional file 1: Figure S7, Additional file 1: Supplementary Results).

## Discussion

We show that polygenic risk burden for RA and CAD significantly impact microglial count density, in different regions and at different stages of activation, in postmortem brain of elderly individuals. However, these associations were driven by only one or two variants within each GRS, highlighting a key limitation in the use of polygenic risk models of complex traits. In joint analyses of GRS, microglial densities, and RNA sequencing from the frontal cortex, we found no significant direct associations between GRS and immune gene module expression. However, when evaluating pleiotropy among GRS variants, microglial density and immune gene expression, we noted significant associations of MS, CAD, and RA risk variants with brain-wide microglial density and expression of at least one immune module. In parallel, a high genetic MS burden was linked to a loss of modules that are enriched for neuronal or mitochondrial genes, suggesting that it may play a role in exacerbating the dysfunction or loss of neurons or their transcriptional programs.

Several GRS tested showed effects in the directions expected based on existing literature: both the AD^+*APOE*^ and AD^-*APOE*^ GRS were significantly associated with cognitive decline at corrected thresholds, but, interestingly, the AD^-*APOE*^ GRS was only significantly associated with tau-related neuropathology, complimenting existing evidence from in vivo PET imaging and CSF analyses showing an effect of *APOE* ε4 on amyloid- but not tau-related biomarkers in healthy elderly [35]. While our observed association of the CAD GRS with microglial density in the inferior temporal cortex was not expected, the variant driving this association, rs4977574, is in high linkage disequilibrium (r^2^ = 0.89) with another variant, rs1333049, that has been associated at genome-wide significance with risk for ischemic stroke [36]. It is possible that the overlapping susceptibility at this locus for CAD and stroke drives cerebrovascular changes that lead to the recruitment and activation of microglia. Analyses of regional interactions between microglia density and other types of brain pathology, such as silent infarction, is beyond the scope of our current study and is a topic of future interest. For our observed association of the RA GRS with microglia across multiple activation stages, there appears to be little regional specificity other than that the association is much less pronounced is the inferior temporal gyrus. Interestingly, this region is the earliest affected in AD [37] and appears to behave differently from the other three brain regions in our analyses: it harbors additional, significant (CAD) and suggestive (MS and AD) associations in secondary analyses, particularly in relation to the activated, stage 3 microglia. Finally, we note that, while we elected to use an FDR-based correction in our original analysis plan, our main results also meet more conservative thresholds of significance, such as Bonferroni correction.

Many tests revealed a lack of GRS effects on our outcomes: for example, the schizophrenia GRS was not associated with any measure of cognitive decline or neuropathology. This seemingly contradicts previous evidence of increased HLA-DR^+^ microglia in brains of schizophrenia patients compared to age-matched controls [38]. Our lack of association of schizophrenia GRS with microglial density in any area and at any stage of activation suggests that the mechanism behind aberrant microglial recruitment in the schizophrenia brain is less likely to be due to schizophrenia-specific genetic risk factors; rather, it may be driven by environmental factors or be a consequence of processes downstream of the onset of schizophrenia. In addition, for telomere length variants, we find a lack of association with AD pathology or cognitive decline, in contrast to a recent meta-analysis finding that AD patients have shorter telomeres than controls [39], as well as Mendelian randomization analyses suggesting causal links between telomere length and AD [40]. Also, our top signals for association across all GRS (including RA) localized to the MHC region, which influences many immune traits, and resolving the mechanism of this association to the HLA class II region within the MHC will be difficult given the extensive linkage disequilibrium that exists in this unique genomic region [41].

The main limitation of our GRS is that they were derived from lists of genome-wide significant loci only. We chose this approach due to (1) the lack of unrestricted availability of full summary statistics for all diseases tested, and (2) validity assumptions of individual variant analyses. If many variants well below genome-wide significance from each GWAS were included in our GRS calculations, then post-hoc associations of individual variants may not be relevant in the context of risk for the disease of interest. Moreover, our post-mortem measure of microglial activation is based on morphologic criteria and is subject to error associated with misclassification of individual cells to specific microglial stages of activation. However, by analyzing multiple binned groups of microglia by stage in our detailed analyses, the confounding of misclassification over the spectrum of groups has likely been mitigated. Also, the challenge of objectively classifying microglial activation states is not unique to our study; microglial staging is an active field of investigation [42].

## Conclusions

Together, our findings demonstrate limited links between microglial activation and liability for archetypal inflammatory diseases of both the central nervous system (MS) and periphery (RA). Thus, the immune dysfunction involved in AD susceptibility seems to be largely distinct from those genes and pathways that are involved in susceptibility to inflammatory disease in young and middle-aged adults. Nonetheless, we have uncovered a handful of variants that have strong effects on both inflammatory disease risk and microglial density, which informs our understanding of human microglial biology in aging which remains poorly understood today.

## Additional file

Additional file 1: Supplementary Methods, Results, and Figures. (DOCX 1972 kb)

## Abbreviations

AD: Alzheimer’s disease
Aβ: Amyloid beta
CAD: Coronary artery disease
CSF: cerebrospinal fluid
eQTL: Expression quantitative trait locus/loci
FDR: False discovery rate
FPKM: Fragments per kilobase per million reads
GRS: Genetic risk score
GWAS: Genome-wide association study
HLA: Human leukocyte antigen
MAP: Memory and Aging Project
MHC: Major histocompatibility complex
MS: Multiple sclerosis
PD: Parkinson’s disease
PET: Positron emissions tomography
PMI: Postmortem interval
RA: Rheumatoid arthritis
ROS: Religious Orders Study
