# Supplementary material for: Polygenic analysis of inflammatory disease variants and effects on microglia in the aging brain
Source: Mol Neurodegener. 2018 Jul 24;13:38. doi: 10.1186/s13024-018-0272-6 (PMC6057096; doi:10.1186/s13024-018-0272-6)
Supplement: Supplementary file 1 — Supplementary Methods, Results, and Figures. (DOCX 1972 kb) [file 13024_2018_272_MOESM1_ESM.docx]

# **Supplementary Methods**

## **Genotype data Quality Control and MHC Region Imputation**

To minimize population admixture, only self-declared non-Latino Caucasians from the Religious Orders Study (ROS) and Memory and Aging Project (MAP) were genotyped. After quality control of the initial genotype dataset, 1 709 individuals and 750 173 autosomal variants remained. The SNP2HLA pipeline [1] uses BEAGLE software and the Type 1 Diabetes Genetics Consortium [2] (T1DGC) reference panel (n_subjects_=5 225, n_variants_=8 961) to impute genetic variation in the major histocompatibility complex (MHC) region (range from chr6:29 494 897- 33 160 425 [hg19 coordinates]) corresponding to SNPs, amino acid substitutions, insertion/deletions, and classical human leukocyte antigen (HLA) alleles at class I and class II loci. After whole genome and MHC-specific imputation, datasets of best-call genotype outputs from both pipelines were merged to allow for genome-wide genetic risk score (GRS) calculations. To provide measures of population stratification, EIGENSTRAT was used to generate principal components from a genotype covariance matrix[3].

## **Postmortem Microglia Quantification**

Immunohistochemistry for microglia was performed using an Automated Leica Bond immunostainer (Leica Microsystems Inc., Bannockborn IL) and anti-human HLA-DP, DQ, DR antibodies (clone CR3/43; DakoCytomation, Carpinteria CA; 1:100) using standard Bond epitope retrieval and detection. An investigator blinded to the clinical and pathologic data, outlined the cortical or subcortical gray region of interest on each slide using a Microbrightfield Stereology System. The Stereo Investigator 8.0 software program was used to place a 1000 × 750μm sampling grid over the region and the program was engaged to sample 4.0% of the region with a 200 × 150μm counting frame at 400x magnification at interval grid intersection points. Using separate tags for stage 1, 2 and 3 microglia, the operator marked the microglia at each intersection point. These counts were then upweighted by the stereology software to estimate total number of microglia (stage 1, 2 and 3) in the defined area. Different stages of activation from least (stage 1) to most (stage 3) activated can be defined morphologically; when these cells become activated, their long fine processes contract and thicken and the cell body adopts a larger more rounded cellular conformation. Data from the two adjacent blocks of tissue (0.5 to 1.0 cm apart) were averaged to obtain composite average densities of microglia in each region.

## **Postmortem amyloid-β and tau pathology**

Quantitative scores for Aβ (percent area occupied) and paired helical filament tau deposition (density) and neuritic and diffuse plaques and neurofibrillary tangles(density by number/mm^2^ in region with highest density) were computed separately and square root-transformed before analysis, as previously described [4,5].

## **RNA sequencing and post-processing**

RNA was extracted from the gray matter of dorsolateral prefrontal cortex using Qiagen's miRNeasey mini kit and the RNase free DNase Set. RNA sequencing (RNA-Seq) was performed on the Illumina HiSeq (50 million paired-end reads of 101 bp each) for samples with a RNA integrity score of greater than 5 and a quantity threshold of greater than 5ug. The RNA-Seq library was prepared on the Broad Institute’s Genomics Platform using strand specific dUTP method [6] with poly-A selection [7]. The SpeakEasy algorithm - used for clustering genes into co-expressed, functionally cohesive modules - combines bottom-up and top-down approaches to clustering, and outperforms other commonly used methods, such as WGCNA[8], on benchmark and real world datasets.[9]

## **Statistical Analysis**

In the full genomic sample (n=1 709), Spearman rank correlations between polygenic scores were evaluated to ascertain relationships between genetic burden for each analyzed trait.

The method of robust regression was chosen to improve coefficient estimation in the presence of any distributional outliers with respect to study outcomes.

To determine variant effects on gene module expression, individual variants from each GRS with minor allele frequency > 0.1 were tested using general linear models, co-varying for important technical and demographic factors.

To assess the significance of correlation rankings in the combined analyses of variant effects on microglial density and gene module expression, the following formula (1) was used to calculate one-sided *P*-values:

$\text{P}_{\text{r}}\text{=}\left(\frac{\text{r}}{\text{n-4}}\right)\text{×}\left(\frac{\text{r+1}}{\text{n-3}}\right)\text{×}\left(\frac{\text{r}+2}{\text{n-2}}\right)\text{ ×}\left(\frac{\text{r}+3}{\text{n-1}}\right)\text{×}\left(\frac{\text{r}+4}{\text{n}}\right)$ (1)

Where n= number of total modules (47), and r = lowest observed immune module rank.

The null hypothesis of this test is that the lowest observed rank for five immune modules out of 47 total modules would be seen due to random ranking assignment. **Supplementary Figure2** shows the distribution of *P*-values as a function of lowest module rank as well as where the observed rheumatoid arthritis and multiple sclerosis GRS ranks fall on the spectrum.

# **Supplementary Results**

## Expression-QTL analyses of SNPs associated with microglial density

In ROS/MAP, the top variant influencing microglial density for the rheumatoid arthritis score (rs9268839, *P*_uncor_=2.48x10^-5^) was significantly associated with *HLA-DQB1* expression in postmortem dorsolateral prefrontal cortex (*P*_uncor_<1x10^-15^, *P*_corrected_<0.0001). According to the GTEx database,[10] rs9268839 is also strongly associated with the expression of *HLA-DRB6* (*P*=6.11x10^-15^) and *HLA-DQB1* (*P*=7.77x10^-7^) in postmortem frontal cortex, Brodmann Area 9 (part of the dorsolateral prefrontal cortex), among many other tissue types.

To evaluate the intersection of gene variant effects on both microglial density and gene expression, Spearman correlations of variant effects on both outcomes were evaluated within each GRS. Again, no results were significant after correction; however, 17 variant effect correlations were significant at *P*_uncor_<0.05 (**Supplementary Figure 7A**). Of these, 15 were for the variants contributing to the MS GRS. As seen in **Supplementary Figure 7**, the five immune modules are among those yielding the strongest positive correlations, while the strongest negative correlations are enriched for neuronal modules[11]. Assessing this statistically, the five immune modules ranked 46^th^, 45^th^, 44^th^, 42^nd^, and 35^th^ out of all 47 when modules are ordered from greatest negative effects correlation to most positive. The probability of observing this skewed ranking (i.e. the lowest rank of five modules being at least 35^th^, if the distribution of associations was random) is 8.4x10^-4­^ (**Supplementary Figure 2**). For the RA GRS, immune gene modules also grouped in high ranks (lowest rank=37^th^, *P*_uncor_=3.0x10^-4^), but no other groups of variants significantly influenced both microglial density and immune-specific gene module expression (**Supplementary Figure 6**).

# **Supplementary References**

1. Jia X, Han B, Onengut-Gumuscu S, Chen W-M, Concannon PJ, Rich SS, et al. Imputing Amino Acid Polymorphisms in Human Leukocyte Antigens. PLOS ONE. 2013;8:e64683.

2. Rich SS, Concannon P, Erlich H, Julier C, Morahan G, Nerup J, et al. The Type 1 Diabetes Genetics Consortium. Ann N Y Acad Sci. 2006;1079:1–8.

3. Price AL, Patterson NJ, Plenge RM, Weinblatt ME, Shadick NA, Reich D. Principal components analysis corrects for stratification in genome-wide association studies. Nat Genet. 2006;38:904–9.

4. Bradshaw EM, Chibnik LB, Keenan BT, Ottoboni L, Raj T, Tang A, et al. CD33 Alzheimer’s disease locus: altered monocyte function and amyloid biology. Nat Neurosci. 2013;16:848–50.

5. Yu L, Chibnik LB, Srivastava GP, Pochet N, Yang J, Xu J, et al. Association of Brain DNA methylation in SORL1, ABCA7, HLA-DRB5, SLC24A4, and BIN1 with pathological diagnosis of Alzheimer disease. JAMA Neurol. 2015;72:15–24.

6. Levin JZ, Yassour M, Adiconis X, Nusbaum C, Thompson DA, Friedman N, et al. Comprehensive comparative analysis of strand-specific RNA sequencing methods. Nat Methods. 2010;7:709–15.

7. Adiconis X, Borges-Rivera D, Satija R, DeLuca DS, Busby MA, Berlin AM, et al. Comprehensive comparative analysis of RNA sequencing methods for degraded or low input samples. Nat Methods [Internet]. 2013 [cited 2015 Jun 8];10. Available from: http://www.ncbi.nlm.nih.gov/pmc/articles/PMC3821180/

8. Langfelder P, Horvath S. WGCNA: an R package for weighted correlation network analysis. BMC Bioinformatics. 2008;9:559.

9. Gaiteri C, Chen M, Szymanski B, Kuzmin K, Xie J, Lee C, et al. Identifying robust communities and multi-community nodes by combining top-down and bottom-up approaches to clustering. ArXiv150104709 Phys Q-Bio [Internet]. 2015 [cited 2015 Sep 13]; Available from: http://arxiv.org/abs/1501.04709

10. GTEx Consortium. Human genomics. The Genotype-Tissue Expression (GTEx) pilot analysis: multitissue gene regulation in humans. Science. 2015;348:648–60.

11. Mostafavi S, Gaiteri C, Sullivan S, White CC, Xu J, Taga M, et al. A molecular network map of the aging brain implicates INNPL1 and PLXNB1 in Alzheimer’s disease. Rev. 2017;

# Supplementary Figures

#
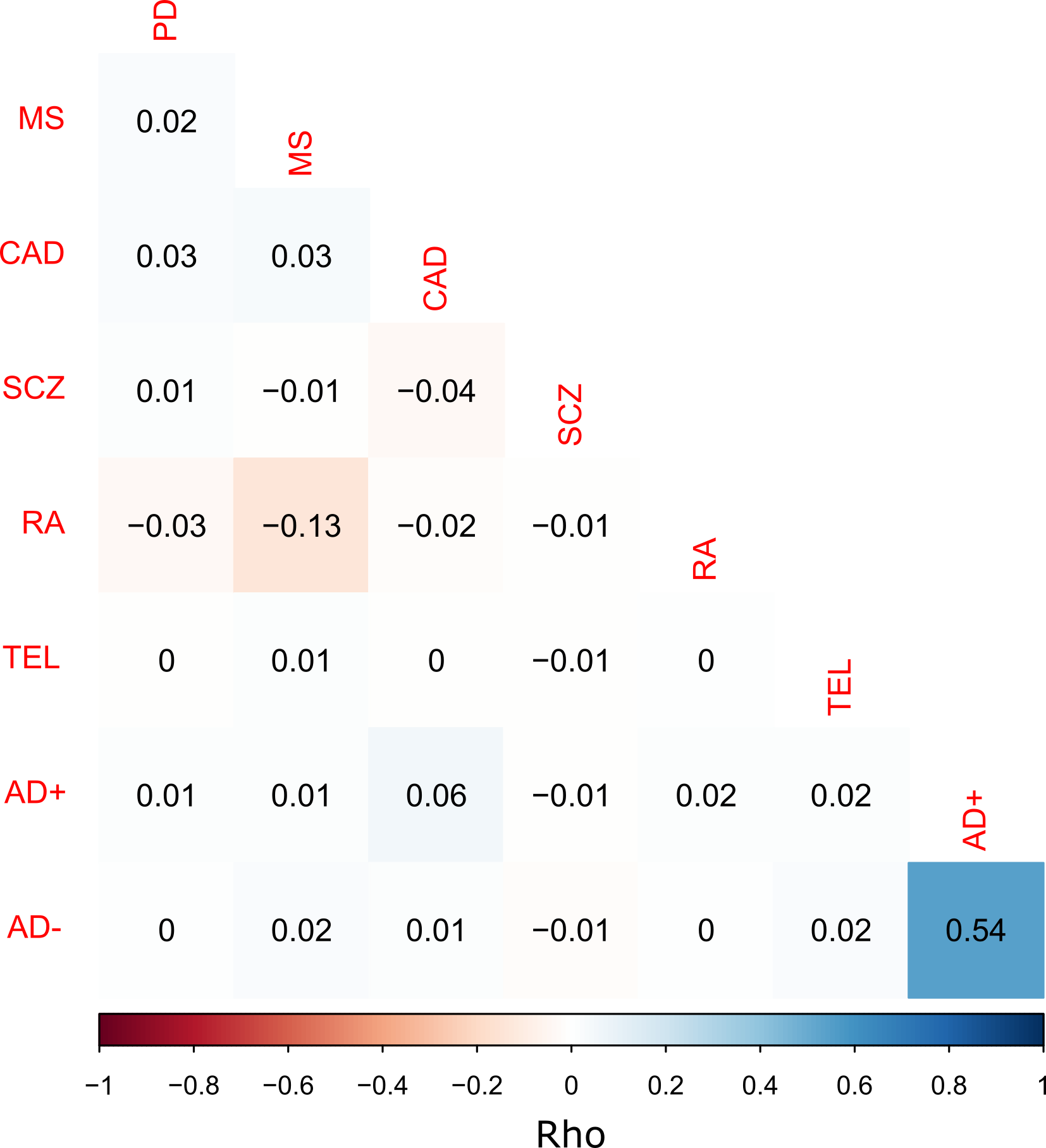


**Supplementary Figure 1. Spearman correlations between each polygenic score (PS)**. AD+ and AD- are Alzheimer’s disease GRS including and excluding APOE, respectively. MS = multiple sclerosis. coronary artery disease = coronary artery disease. SCZ = schizophrenia. rheumatoid arthritis = rheumatoid arthritis. TEL = telomere length.

#
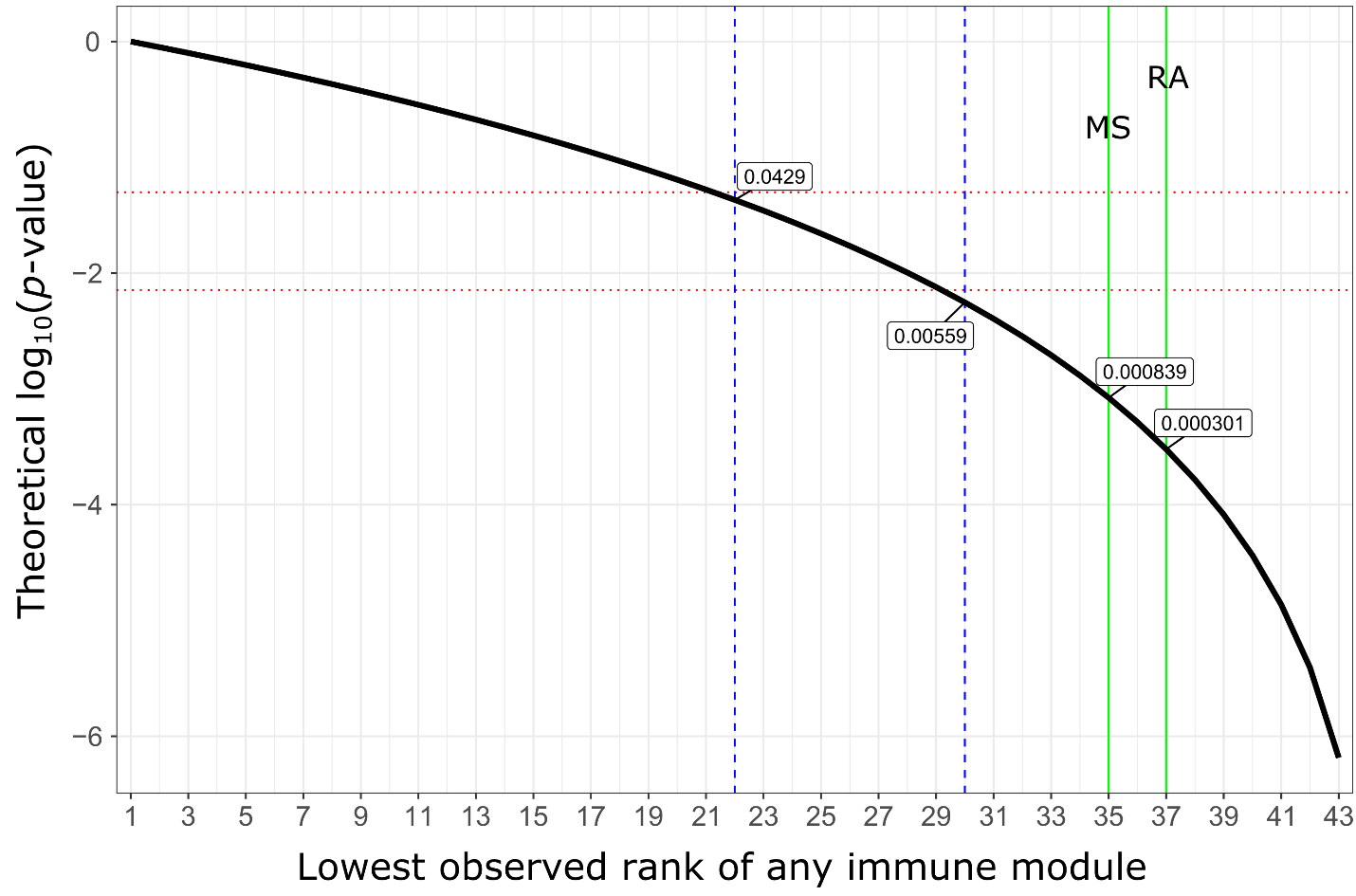


**Supplementary Figure 2. Probability of observing rank clustering in variant effect correlation analyses.** The green lines represent the lowest observed rank for rheumatoid arthritis (RA) and multiple sclerosis (MS) scores. The dotted red and blue lines indicate the lowest observed rank of any immune gene module at uncorrected (lowest rank = 22, P=0.043) and Bonferroni corrected (lowest rank = 30, P=0.0056 (0.05/7)) levels of significance.


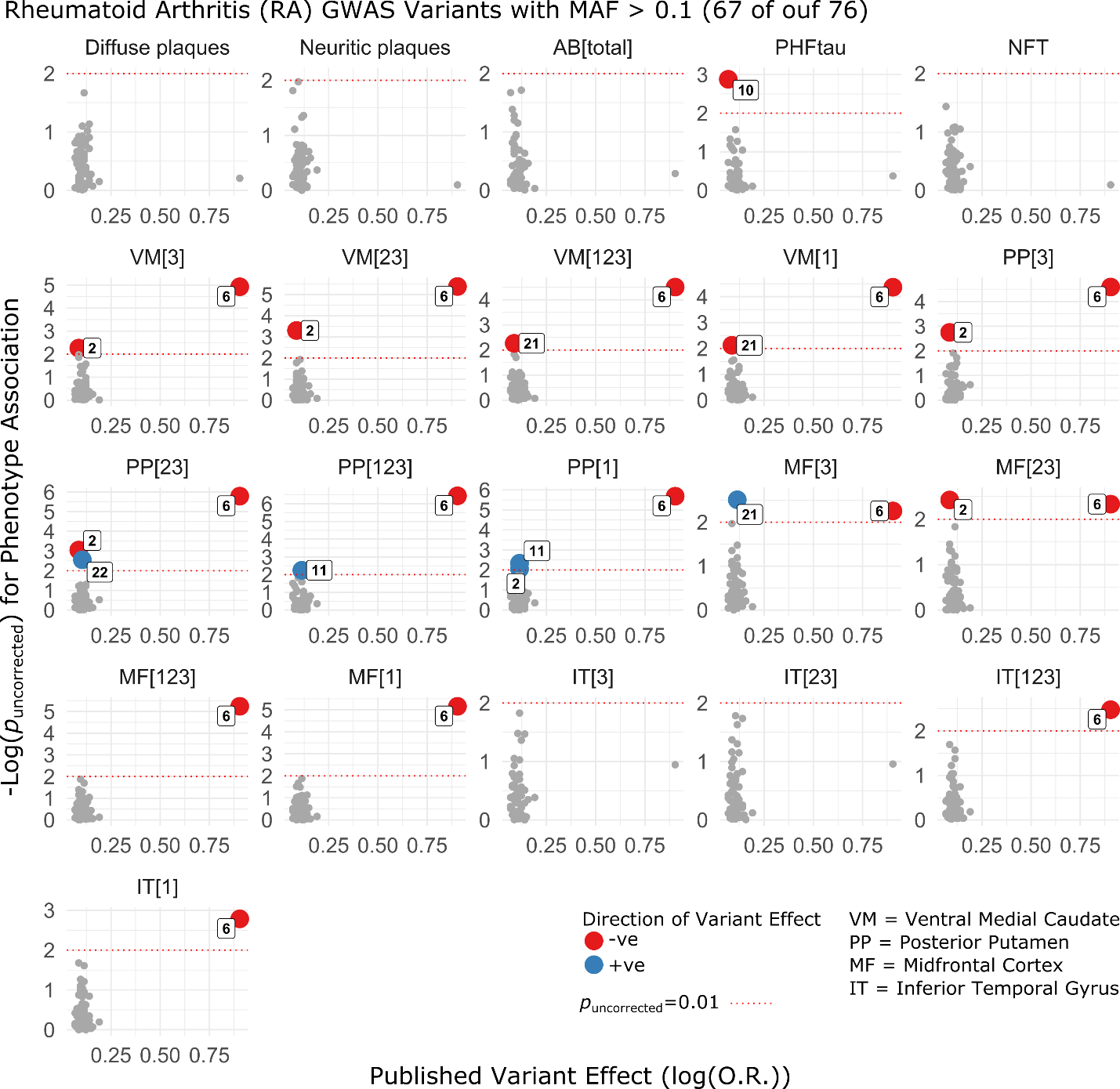


**Supplementary Figure 3.** **Associations of individual variants in the rheumatoid arthritis (rheumatoid arthritis) polygenic score on all postmortem pathological outcomes.** Published effect sizes on the x-axis have been transformed using a natural logarithm and oriented in the positive direction to align allelic effects (color denotes direction of effect on microglial density). Microglial density phenotypes are indicated by region acronym and activation stage number (in square brackets). P-values are two-sided and derived from robust iterated re-weighted least squares regression models, co-varying for age at death, sex, and three EIGENSTRAT principal components. The red dotted lines indicate uncorrected P=0.01. PHFtau = paired helical filament tau. NFT = neurofibrillary tangles. VM = ventral medial caudate. PP = posterior putamen. MF = midfrontal cortex. IT = inferior temporal cortex.


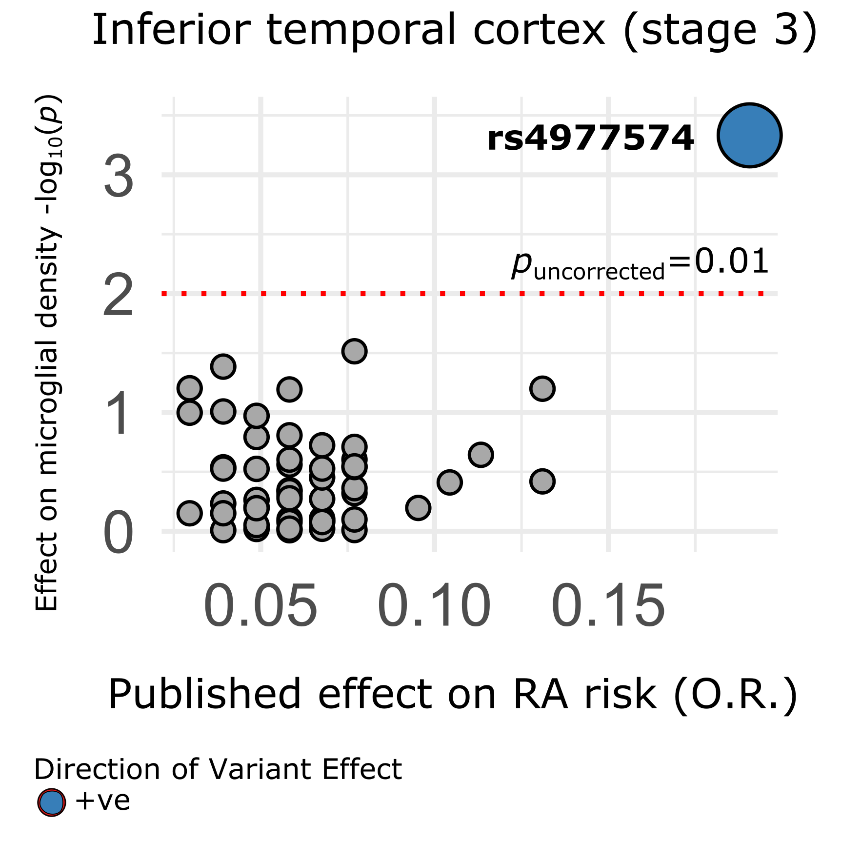


**Supplementary Figure 4. Analysis of individual variants in the coronary artery disease polygenic score on microglial density in the inferior temporal cortex.** Published effect sizes on the x-axis have been transformed using a natural logarithm and oriented in the positive direction to align allelic effects (color denotes direction of effect on microglial density). P-values are two-sided and derived from robust iterated re-weighted least squares regression models, co-varying for age at death, sex, and three EIGENSTRAT principal components.


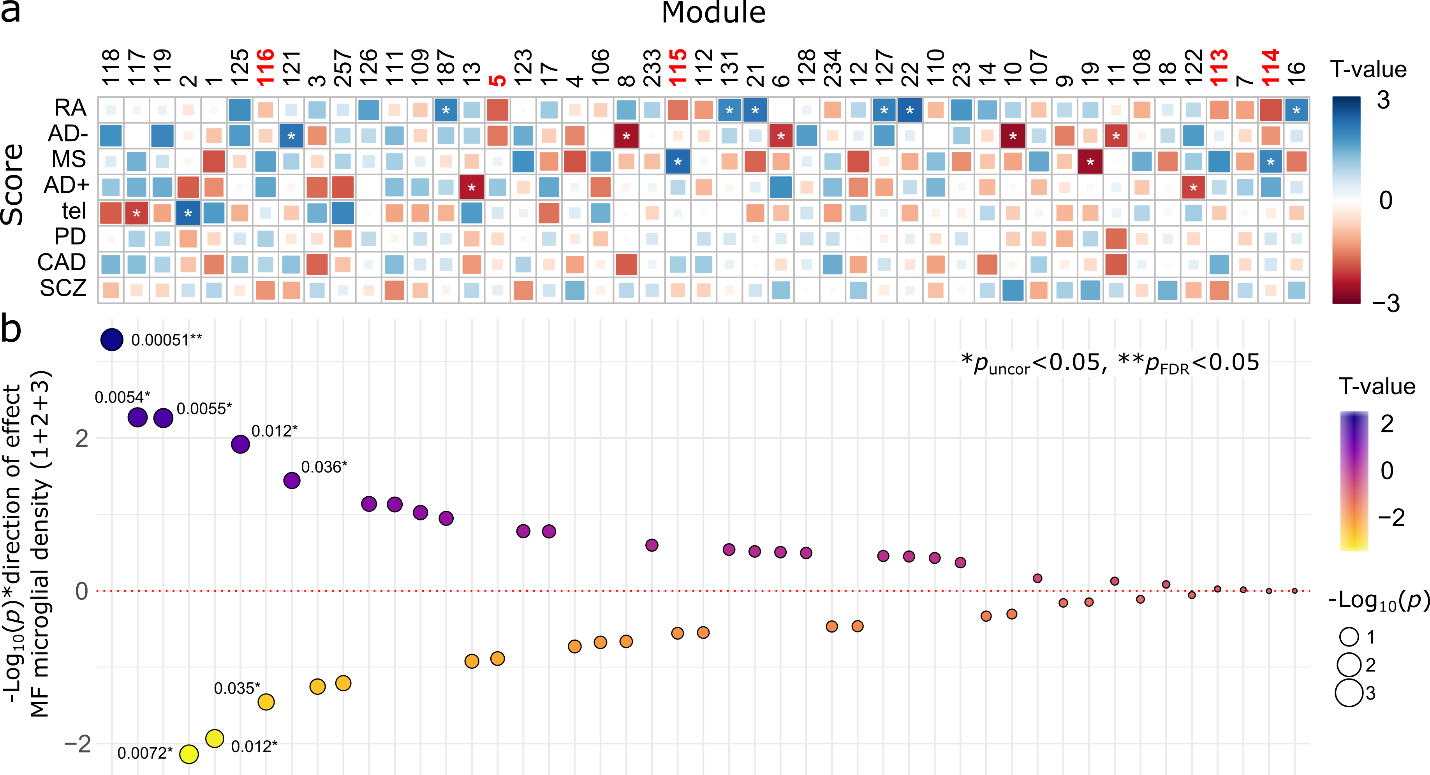


**Supplementary Figure 5. Associations between gene module expression and A) GRS and B) midfrontal (MF) microglial density.** Modules are ordered left to right by ascending P-value for associations of variants with MF microglial density at stages 1+2+3. Polygenic scores in panel A are ordered top to bottom by descending number of significant associations with gene module expression at P_uncorr_<0.05. T statistics were derived from robust regression, and P-values are two sided and uncorrected. AD+ and AD- are Alzheimer’s disease GRS including and excluding APOE, respectively. MS = multiple sclerosis. coronary artery disease = coronary artery disease. SCZ = schizophrenia. rheumatoid arthritis = rheumatoid arthritis. TEL = telomere length.


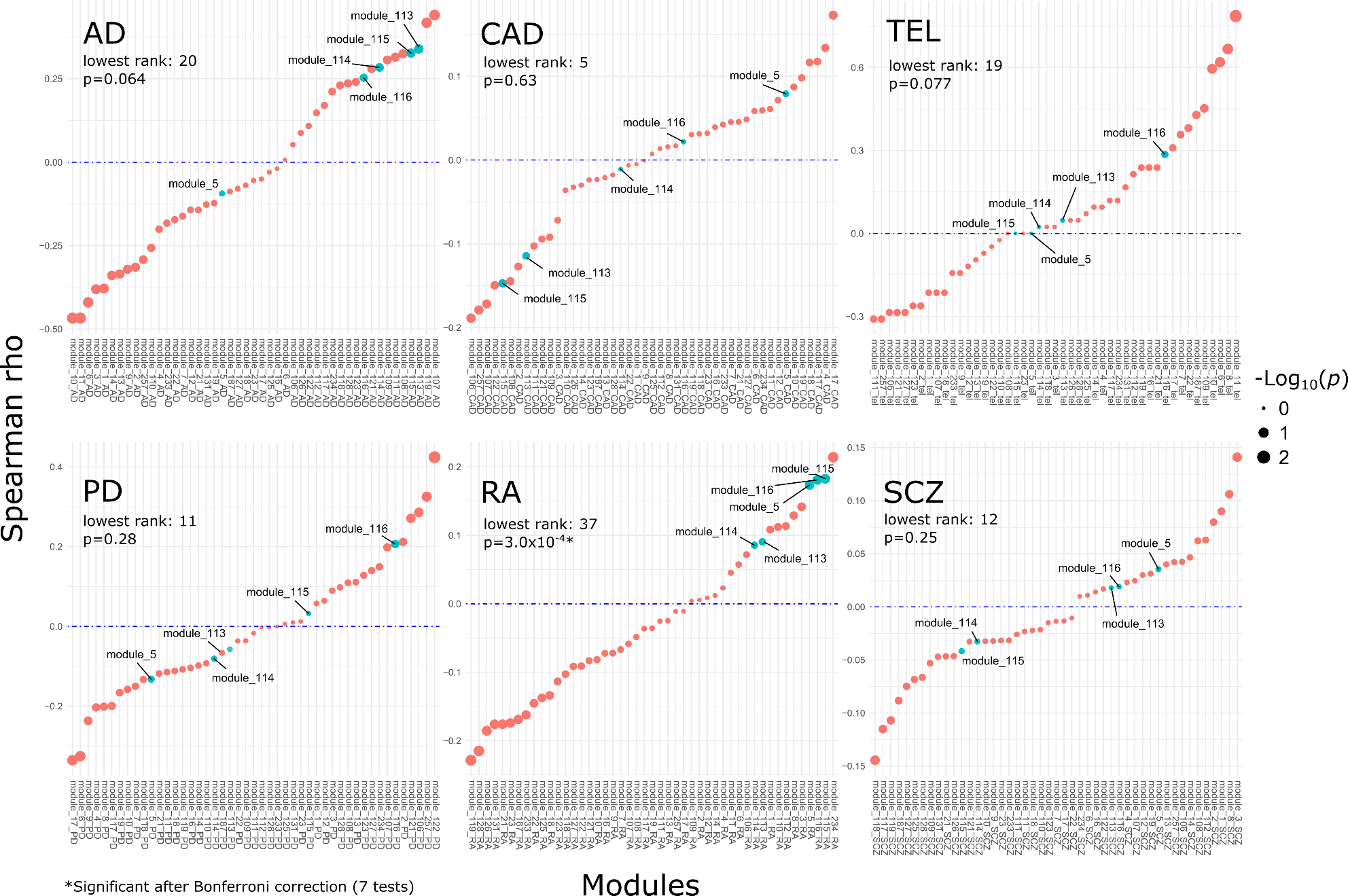


**Supplementary Figure 6. Analysis of shared effects of gene variants on microglial density and gene module expression.** Spearman correlations between gene variants’ effects on microglial density vs. their effects on gene module expression for each GRS other than multiple sclerosis (MS). Modules are ordered separately for each GRS per the strength of correlation between the effects of variants in each score on both outcomes. Immune modules are indicated and p-values indicate the probability of observing the clustering of all five immune modules above the lowest observed rank, and are therefore one-sided. The Alzheimer’s disease GRS in this analysis included effects of APOE. coronary artery disease = coronary artery disease. SCZ = schizophrenia. rheumatoid arthritis = rheumatoid arthritis. TEL = telomere length.


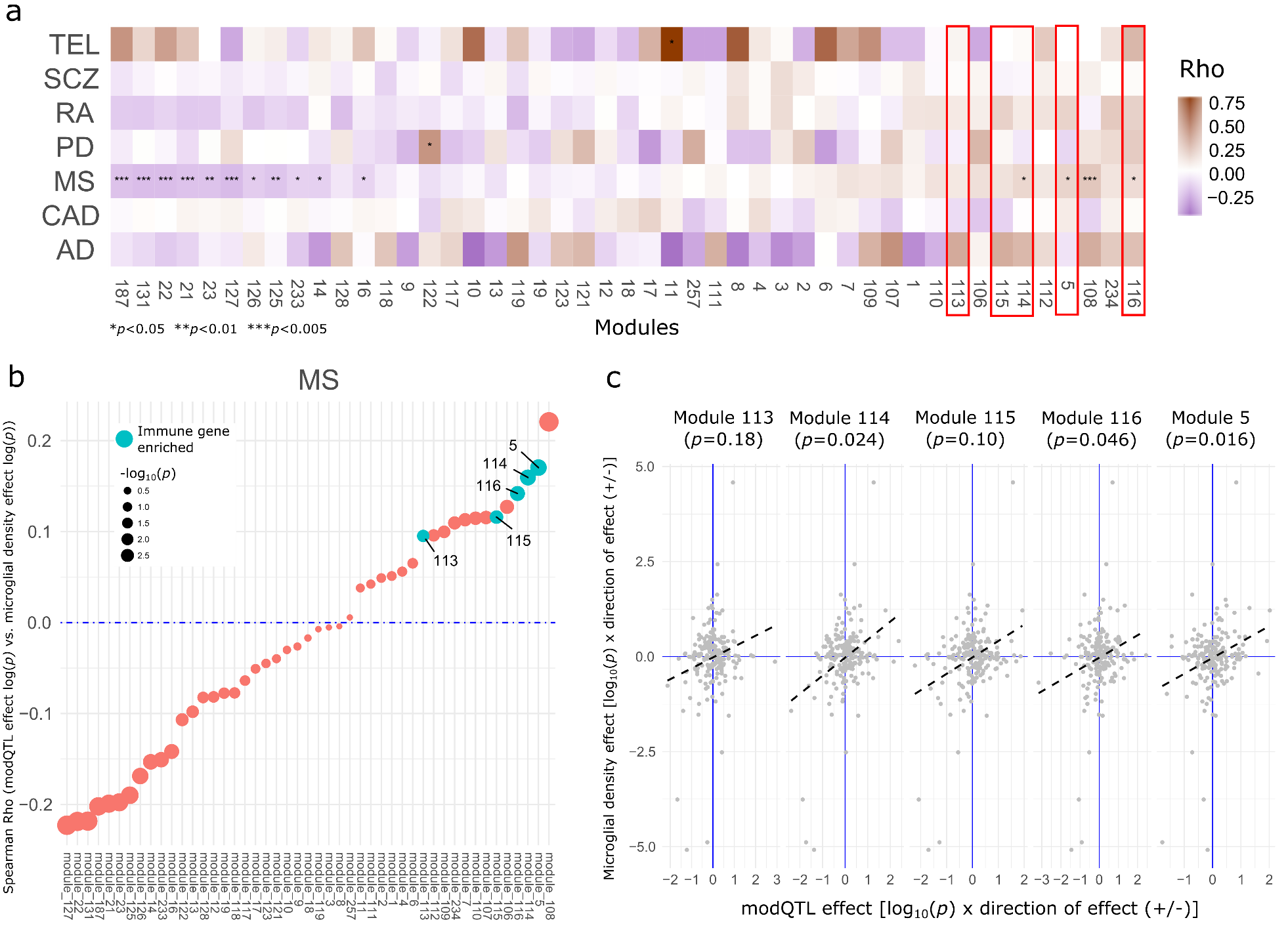


**Supplementary Figure 7**. **Analysis of shared effects of gene variants on microglial density and gene module expression.** A) Spearman correlations between gene variants’ effects on microglial density vs. their effects on gene module expression for each GRS and 47 modules, ordered by effect correlation among all variants combined (increasing left to right). The AD GRS in this analysis included effects of *APOE*. B) Spearman correlations for variants only within the MS GRS, ordered by increasing rho (left to right), where significance of the correlation is indicated by point size. C) Correlations of MS variant effects on aggregate brain-wide microglial density (y-axis) vs. MS variant effects on module expression of five immune modules, on a scale of significance (-log_10_(P)) weighted by correlation coefficient sign. Note that the lines of best fit shown do not represent the rank order statistical test performed and are intended to show direction of effect only. P-values are two-sided and describe the significance of Spearman’s ρ for each correlation.
